# Accelerometry-Based Classification of Human Activities Using Markov Modeling

**DOI:** 10.1155/2011/647858

**Published:** 2011-09-04

**Authors:** Andrea Mannini, Angelo Maria Sabatini

**Affiliations:** Scuola Superiore Sant' Anna, Piazza dei Martiri della Libertà 33, Pisa 56125, Italy

## Abstract

Accelerometers are a popular choice as body-motion sensors: the reason is partly in their capability of extracting information that is useful for automatically inferring the physical activity in which the human subject is involved, beside their role in feeding biomechanical parameters estimators. Automatic classification of human physical activities is highly attractive for pervasive computing systems, whereas contextual awareness may ease the human-machine interaction, and in biomedicine, whereas wearable sensor systems are proposed for long-term monitoring. This paper is concerned with the machine learning algorithms needed to perform the classification task. Hidden Markov Model (HMM) classifiers are studied by contrasting them with Gaussian Mixture Model (GMM) classifiers. HMMs incorporate the statistical information available on movement dynamics into the classification process, without discarding the time history of previous outcomes as GMMs do. An example of the benefits of the obtained statistical leverage is illustrated and discussed by analyzing two datasets of accelerometer time series.

## 1. Introduction

Many technical applications could greatly benefit from the availability of systems that are capable of automatically classifying specific physical activities of human beings. In this paper, either static posture, for example, standing, or dynamic motion, for example, walking, is included in the term physical activity. The sort of contextual awareness coming from this knowledge [1] may help improving the performance of healthcare monitoring devices or promoting the development of advanced human-machine interfaces. In fact, the precise activity performed by the subject helps defining the context in which further estimation can be conducted. Consider, for instance, the problem of estimating the metabolic energy expenditure of a human subject by indirect methods [2]: these methods are reported to incur severe estimation errors in the absence of any information about the particular functional task in which the subject is actually involved [2, 3]. In robotics, several applications, notably in rehabilitation engineering, demand some capability by the robot controller of recognizing the user's intent. In particular, smart walking support systems have been developed to assist motor-impaired persons and elderly in their efforts to stand and to walk [4, 5] or to detect gait instabilities of the user [6, 7] and minimize the risk of fall [7].

In principle, the wearable sensors needed to elicit the contextual information would be characterized by low power consumption, small size and weight, and adequate metrological specifications. Microelectromechanical systems (MEMS) motion sensors appear well matched to these requirements [8]. The methods investigated in this paper revolve around the processing of acceleration signals acquired from small networks of MEMS accelerometers positioned at few anatomical points of the human body. The machine learning algorithms considered in this paper are a useful complement to the computational methods that are used for pose estimation and navigation using inertial sensors [9].

A major part of this paper consists of illustrating and discussing an approach for classification of human physical activities, which is based on using Hidden Markov Models (HMMs). In principle, this approach aims at exploiting the information available on the movement dynamics, namely, the capability of recognizing activities performed at the current time is related to the classification outcomes provided in the past by the classifier. Accordingly, we talk about sequential classifiers, which differ from the so-called single-frame classifiers in the sense that the latter are interested in single activity primitives, in other words, elementary activities are studied in isolation from the history of previously detected activities [9–16]. 

Nowadays, HMMs find applications in a large number of recognition problems, including, but not limited to, speech recognition [17], hand gesture and sign language recognition [18], and controlling robotic tools by hand gesture [19]. Concerning the human activity recognition, most studies on the application of HMMs [20, 21] are based on camera recordings, as shown by Yamato et al. in [22], although few research reports are now aiming at applications of HMMs to collect data from wearable sensor systems [23, 24]. These studies focus on the validation of statistical models of each considered activity. In a different way, our approach is based on using lightweight wearable sensors and is oriented to exploit HMMs at a higher level. In particular, their use can be oriented towards modeling time relations between elements of a sequence of activities. Few applications of HMMs are reported in the literature as for the problem of classifying human physical activities from inertial sensors, probably because HMMs are known to be potentially plagued by severe difficulties of parameter estimation. In this paper we propose a way of alleviating this difficulty by adopting a supervised approach to classifier training. This approach is feasible when the data available in the training set are annotated. 

## 2. Materials and Methods

### 2.1. Datasets for Physical Activity Classification

The present work is partly based on analyzing the dataset of acceleration waveforms published by Bao and Intille [10]; the dataset, henceforth referred to as the B&I dataset, was released to us by the authors. Acceleration data, sampled at 76.25 Hz, are acquired from five biaxial accelerometers, located at the hip, wrist, arm, ankle, and thigh. The original protocol is based on testing 20 subjects, who are requested to perform 20 activities of daily living. These include activities that primarily involve the use of the upper limbs (e.g., brushing teeth, scrubbing, folding laundry, eating or drinking, and working on computer), whole-body activities (e.g., stretching, and vacuuming), and activities that primarily involve the use of the lower limbs (e.g., walking, climbing stairs, running, and cycling). In addition, static postures are considered (e.g., standing still, sitting and relaxing, lying down and relaxing) together with quasi-static postures (e.g., reading, watching TV). The B&I dataset include, for each subject, acceleration time series that are known to correspond to a specific activity performed by her/him. We refer to any activity as an *activity primitive,* to distinguish it from higher-level activities, namely, motor behaviors that result from chaining several activity primitives in some temporal order. Acceleration signals related to higher-level activities are not available in the B&I dataset, since the research goal in [10] is exclusively to test single-frame classifiers.

**Figure 1:**
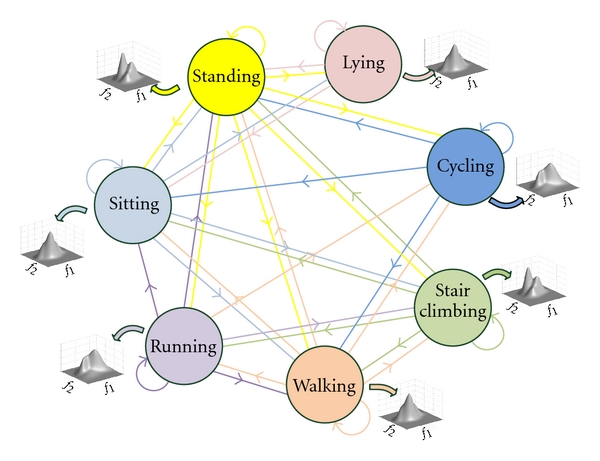
Scheme of a sequential classification based on HMMs.

In this paper, we select the seven activities shown in Figure 1, giving rise to a reduced dataset, henceforth called *seven-activity* dataset. We assume that a sequence of activity primitives, say, an activity at the *motor sentence* level can be modeled using a first-order Markov chain, composed of a finite number *Q* of states *S*_*i*_ (*Q* = 7). Each state accounts for an activity primitive, say, an elementary activity at the *motor word* level: *S*_1_, lying down and relaxing; *S*_2_, cycling; *S*_3_, climbing stairs; *S*_4_, walking; *S*_5_, running; *S*_6_, sitting and relaxing; *S*_7_, standing still. The time evolution of a first-order Markov chain is governed by the vector ***π*** of prior probabilities and the transition probability matrix (TPM) **A**, which helps describing human actions at the behavioral level. The prior probability vector ***π***, with size (1 × *Q*), is composed of the probabilities *π*_*i*_ of each state *S*_*i*_ of being the state *X* at the initial time *t*_0_

(1) $$\pi_{i}=\text{Pr}\left(X\left(t_{0}\right)=S_{i}\right),\;i=1,\ldots ,Q.$$

The elements *a*_*ij*_ of the matrix A, with size (*Q* × *Q*), are the probabilities of transitions from the state *S*_*i*_ at time *t*_*n*_ to the state *S*_*j*_ occupied at time *t*_*n*+1_

(2) $$a_{ij}=\text{Pr}\left(X\left(t_{n}\right)=S_{j}\;|\;X\left(t_{n-1}\right)=S_{i}\right),\;i,j=1,\ldots ,Q.$$

The prior and transition probabilities needed to create the Observable Markov Model (OMM) (***π***, **A**) associated with the Markov chain can be empirically determined based on observations of the activity behavior of a subject. 

In order to overcome the limitations of the B&I dataset when applied to model sequential data, we propose the concept of the virtual experiment. The virtual experiment is a sort of generative model, which allows to concatenate activities together and to simulate complex behaviors mainly for algorithm validation and testing. Simulating a complex behavior by a single subject in our study (virtual experiment) requires that each model state may emit data frames that are randomly sampled (with replacement) from the *N* frames available for the given subject and the activity primitive associated to the emitting state (18 ≤ *N* ≤ 58). Henceforth, data frames are referred to as sliding windows with finite and constant width, whose samples are used to compute the feature vectors needed by the classification algorithm, see Section 2.2. for the seven-activity dataset, the data frames last about 6.7 s (50% overlapping). For each subject, we perform twenty virtual experiments (*S* = 20), each of which is composed of *T* = 300 data frames. The OMM associated with each virtual experiment is built using the TPM specification shown in Table 1.

The procedure of synthesizing virtual experiments in the manner described above implies the existence of clear-cut borders between data frames associated to different primitives, without transients between consecutive data frames that may be unknown to the classifier. This problem is usually managed by manual data cropping in creating the dataset [10]. Of course, real-life data would be more complex and fuzzy, especially for the postural transitions between different activities. In the attempt to get a more realistic picture of the performance achieved by sequential classifier, data frames from the original dataset not included in the reduced dataset are randomly interspersed in the tested data sequences generated by the OMM in proportion 1 : 3. The resulting garbage is managed by the method of rejection of spurious data described in Section 2.5.

The virtual experiment approach we propose is to be considered a useful method for preliminary algorithm validation and testing. At the time being, the wearable system ActiNav is making its first steps in our lab for applications in the field of pedestrian navigation and smart estimation of biomechanical parameters; therefore, it is a welcome addition to the tools we have for investigating the Markov modeling approach to human activity classification. 

**Figure 2:**
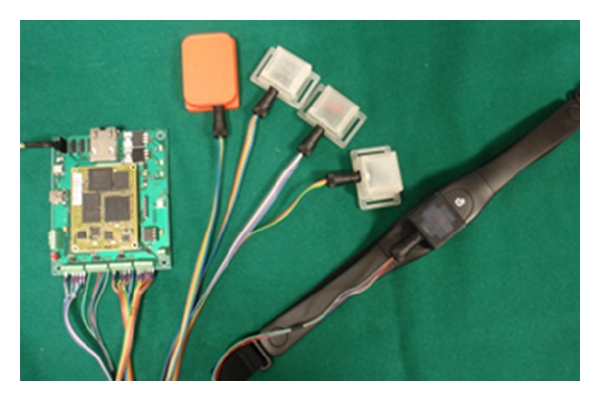
The ActiNav board is shown with several sensors connected to its input ports.

ActiNav revolves around an ARMadeus Board (APF27). It is equipped with an ARM9-based Freescale processor, having 128 MB of RAM, 256 MB of flash memory, and a 200 K-gates Xilinx FPGA. A custom printed circuit board allows arming the APF27 with a 12-bit Successive Approximation Register ADC (AD7490, Analog Devices, Inc.). This converter operates up to 1 MSPS; moreover, since it is endowed with 16 analog channels, up to five triaxis analog accelerometers or gyros can be integrated in ActiNav. The system with the main board (100 × 84 × 16 mm) and different sensors connected is shown in Figure 2. 

For the work described in this paper, a single triaxis accelerometer (ADXL325, Analog Devices, Inc.) with full-scale ±5 g (1 g = 9.81 m/s^2^) is fastened on the right thigh of seven healthy subjects. Each subject performed twenty repetitions of the following task: initially, he sat on a chair, then he stood up, and, after a pause of few seconds, he began to walk. Each trial lasted about 15 s. Sensor data are acquired at a sampling frequency of 250 Hz; moreover, they are manually annotated during acquisition (supervised approach). Specifically, the experimenter attempts to also define the time intervals where the transitions between different activities take place. The data frames are selected to last 250 ms (50% overlapping). This low-complexity dataset, henceforth called the *sit-stand-walk* dataset, allows us to test the proposed methods on a real sequential dataset that includes a postural transition and the incipient locomotion situation. 

### 2.2. Data Processing: Feature Vectors

The automatic classification of acceleration data requires a preprocessing phase in which feature variables with high information content are extracted from the data frames that the measurement channels of the system make available we have ten measurement channels in the* seven-activity* dataset and three measurement channels in the *sit-stand-walk* dataset. 

Following the indications reported in previous works [10, 15], the feature variables considered in this paper are:

- *DC component*. This feature—helpful in discriminating static postures—is evaluated by averaging the raw samples in each data frame. One feature per measurement channel is obtained.
- *Energy*. This feature—helpful in assessing the activity strength—is evaluated as the sum of squared spectrogram coefficients within each data frame. The first coefficient that includes information about the DC component is excluded from the sum. One feature per measurement channel is obtained.
- *Entropy of spectrogram coefficients*. This feature is helpful in discriminating activity primitives that differ in frequency domain complexity [10]. A kernel density estimator is applied to spectrogram coefficients for its determination. One feature per measurement channel is obtained.
- *Correlation coefficients*. These features are the elements of the data covariance matrix; they are computed by applying the dot product to data frames from pairs of measurement channels, provided that the data frames are detrended and normalized to the window size; the correlation coefficients are helpful in discriminating activities that involve motions of various body parts. A total of 55 and 6 correlation coefficients are computed, respectively, for the *seven-activity* and the *sit-stand-walk* datasets.

Before applying the classification algorithm, the feature vectors are selected in order to reduce the dimensionality of the problem, which can be critical especially for the *seven-activity* dataset, where 85 feature variables are computed (15 for the *sit-stand-walk* dataset). Feature selection is required to limit the risk of incurring in severe overfitting [25]. We use the Pudil's algorithm—a sequential forward-backward floating search (SFFS-SFBS) [26]; this algorithm uses the Euclidean distances between each pair of feature vectors of the same class in the training set as a criterion for selecting the most informative feature variables. The criterion for optimizing the feature set derives from a cross-validation study based on a *k*-nearest neighbor classifier (*k*-NN). Iteratively, the Pudil's algorithm modifies the number of features and repeats the validation process. After that, all features have been included once at least in the feature set the feature set of minimal size that maximizes the criterion is selected. A widely used feature extraction method, that is, the *principal component analysis* (PCA), is also applied to feature vectors [25]. 

### 2.3. Single-Frame Classification

Although several single-frame classifiers can be proposed, we consider here a particular technique for single-frame classification, namely, the Gaussian Mixture Model (GMM) classifier. This approach is reported by Allen et al. [9] to achieve very promising results. In particular, the authors discuss the high adaptability of the classifier, a good feature to analyze data from subjects that are not included in the training set. 

Of course, other methods for single-frame classification of human physical activity can be chosen, and they may also outperform GMMs [27]. Here, the GMM classifier is selected as the single-frame classifier of reference, in particular for its resemblance to the structure of an HMM. As a matter of fact, the probability density of emissions of each state in an HMM can be modeled as a Gaussian mixture.

The GMM classifier first performs a parametric estimation of class-conditional probability density functions *p*(**x** | *w*_*i*_), which assign the probabilities of the feature vector **x** given its membership to the class *w*_*i*_. In the training phase, class-conditional probabilities are estimated as Gaussian mixtures. Each feature vector **x** is then classified in the class yielding the highest value of *p*(**x** | *w*_*i*_).

### 2.4. HMM-Based Classification

In modeling sequences of human activities as first-order Markov chains, we propose that the prior and transition probabilities that are associated to the model are empirically determined by observing the subject behavior. If the TPM and the state at the current time are known, then the most likely state that will follow is probabilistically determined. However, each activity primitive can only be observed through a set of raw sensor signals (the measured time series from on-body accelerometers, in the present case). In other terms, the states are hidden and only a second-level process is actually observable (emissions). The statistical model including the pair (***π***, **A**) and the emission process is an HMM. We opt for a continuous emissions approach (*continuous emissions densities *HMM, aka cHMM, [17]). The most common approach to the problem of modeling continuous emissions is parametric. In particular we consider for the *i*-th state *S*_*i*_, namely the class *w*_*i*_, mixtures of *M* multivariate normal distributions *N*(***μ***_*im*_, Σ_*im*_) that are specified by assigning the mean value vectors ***μ***_*im*_, the covariance matrices Σ_*im*_, and the matrix **C** of the mixing parameters *c*_*im*_:

(3) $$p\left(x\;|\;S_{i}\right)=\overset{M}{\underset{m=1}{\sum }}c_{im}N\left(x\;|\;\mu_{im},{\text{Σ}}_{im}\right),\;i=1,\ldots ,Q,$$

where

(4) $$\overset{M}{\underset{m=1}{\sum }}c_{im}=1,\;i=1,\ldots ,Q.$$

The mixture is used to model the emissions from each state in the chain. An excellent reference source for HMMs and algorithms for their learning and testing in a recognition problem is in [17].

We consider a *Q*-state cHMM as represented in Figure 1 for the *seven-activity* dataset, where *Q* = 7 (*sit-stand-walk *dataset: *Q* = 3). A Gaussian cHMM trained in a *d*-dimensional feature space, with *Q* states and *M* components for each mixture, requires the specification of the following parameters:

- ***π***, prior probability vector, 1 × *Q*;
- **A**, transition probability matrix *Q* × *Q*;
- ***μ***, set of mean value matrices, *Q* × *M* × *d*;
- *𝚺*, set of covariance matrices, *Q* × *M* × *d* × *d*;
- **C**, set of mixing parameters, *Q* × *M*.

One of the main problems with this approach may be in the high number of parameters to be identified. The approach to deal with the parameter identification problem we pursue is to split the training phase into two different steps: a first-level supervised training phase is followed by a second-level training phase, which is performed by running the Baum-Welch algorithm [17]. An inaccurate initialization of parameters could easily lead to suboptimal results when using the Baum-Welch algorithm, due to the presence of many local maxima in the optimization surface [17]. However, the training sets turn out to be labeled in the application described in this paper. Therefore, the first level supervised training becomes the proposed way for achieving a good initialization of parameters entering the second level of training.

**Figure 3:**
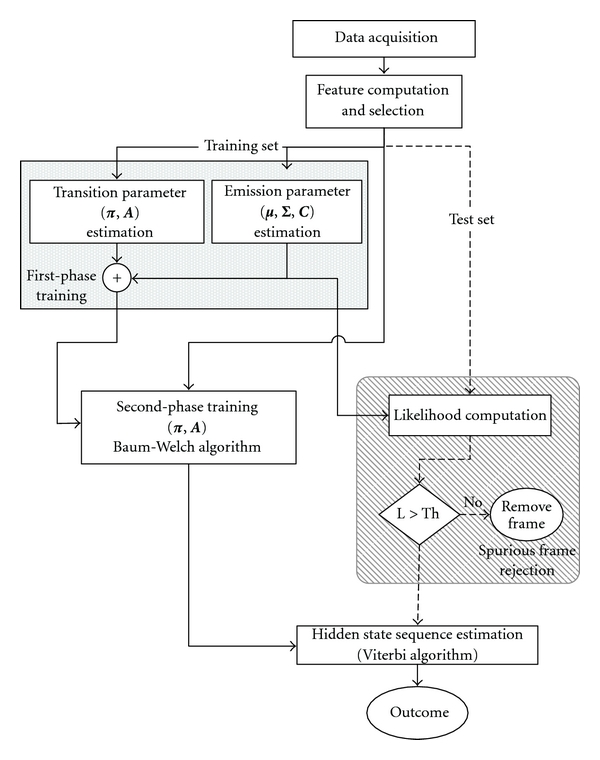
Block diagram of the developed cHMM-based sequential classifier.

In order to simplify the estimation process, the parameter set is divided into two main groups, namely, transition parameters (***π***, **A**) and emission parameters (***μ***, *𝚺*, **C**). This separation allows us to train separately two parameter sets with reduced size, yielding a relevant reduction of the overall size of the training set. Since activity labels from training set examples are assumed to be known, simple methods of counting event occurrences allow us to estimate transition parameters [17]. For instance, the probability *a*_*ij*_ of a transition from the *i*-the state to the *j*-th state is estimated as follows:

(5) $${\hat{a}}_{ij}=\frac{N_{i\to j}}{N},$$

where  *N*_*i*→*j*_  is the number of transitions from the *i*-th state to the *j*-state counted in a training set with size *N*. Emission parameters can be estimated by fitting Gaussian mixture distributions with *M* components to the feature vectors emitted by each state, in a similar fashion to the procedure used to learn GMM classifiers. The training process at the second level exploits the values of the parameters estimated during the training process at the first level, as initial values for running the Baum-Welch algorithm; in our current implementation, this step helps refining the estimates of the transition parameters, Figure 3. In the module for spurious frame rejection, the likelihood L is compared with a suitably chosen threshold Th. The optimal state sequence traced by the cHMM is estimated using a standard Viterbi decoder [17].

A leave-one-out validation study is performed in this paper for both GMM and cHMM-based classifiers. This means that a classifier is trained using data from all subjects but one, and then it is tested on data from the excluded subject only. The cross-validation process is repeated a number of times, each time excluding one different subject from training. Results are then aggregated from the different models. This validation approach allows testing the ability of each classifier to classify correctly new examples that differ from those used for training (generalization); good performances in terms of generalization are essential to prevent the need for individual model calibrations.

### 2.5. Spurious Data Rejection

The strategy of classification we adopt allows us to define a criterion for automatic rejection of spurious feature vectors. If a threshold-based detector is applied to estimated class-conditional probabilities *p*(**x** | *w*_*i*_), it is possible to reject those feature vectors in the classification of which is believed to be too uncertain; we are not forced to introduce additional states, or mixture components, specifically for unknown data. Remind that, in a GMM or a cHMM, *p*(**x** | *w*_*i*_) refers to the probability of the feature vector **x** as the emission of the model state *w*_*i*_. If, for any feature vector, the probabilities relative to each state are below a given threshold, the feature vector itself can be marked as spurious and removed, without affecting the classifier operation. Low values of *p*(**x** | *w*_*i*_) are typical when unknown activities are hidden in the data presented to the classifier or when too much uncertainty affects them. 

**Figure 4:**
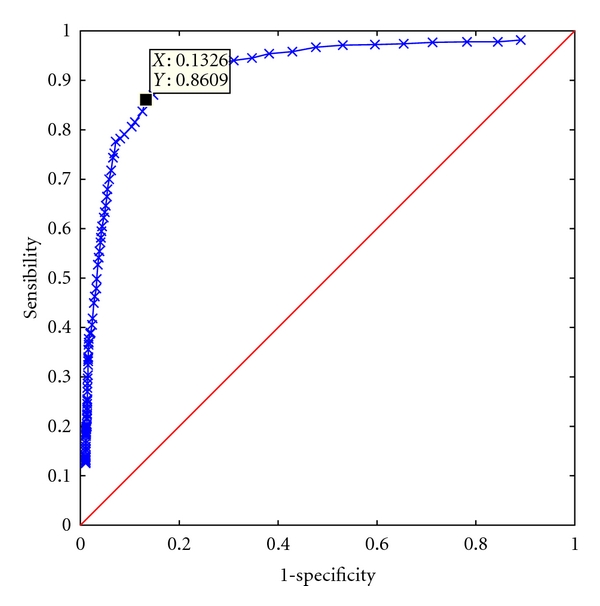
ROC curve obtained using different threshold values.

The threshold value is optimized by studying the specificity-sensitivity curve (ROC curve); averaged across subjects, it is reported in Figure 4 (*seven-activity* dataset). The threshold is chosen when the specificity is slightly greater than the sensitivity.

## 3. Results

We empirically determine whether or not rotation and dimensionality reduction would be jointly pursued when the PCA is applied to the feature variables surviving the Pudil's method. Guided by the results of preliminary testing, we use the PCA cascaded to the Pudil's method for pure rotation in the feature space (*seven-activity* dataset) and for rotation and dimensionality reduction (*sit-stand-walk* dataset). In the former case, thirteen features—all of them being correlation coefficients—are retained for further processing. In the case of the *sit-stand-walk* dataset, three principal components are considered (97.8% of variance is retained), after that the Pudil's method selected seven out of fifteen feature variables. 

The *k*-NN classification accuracies achieved by the Pudil's method are 99.5% and 99.2% (*seven-activity* and *sit-stand-walk* dataset, resp.). It is important to outline that these values have nothing to do with the classification accuracies reported in the following for the GMM and the cHMM-based classifiers. Indeed, the feature selection process is based on a cross-validation study extended to the whole dataset, while classifier testing is based on a leave-one-out approach.

The number of Gaussian components of the mixture is taken *M* = 1, both in the GMM and the cHMM-based classifiers. The experimental evidence does not clearly support the assumption that the data distributions are uni-modal for both datasets; nonetheless, testing up to *M* = 5 does not provide convincing arguments that the simpler choice *M* = 1 would be dismissed. As for the *seven-activity* dataset, Tables 2 and 3 clearly show that *M* = 1 is the winning choice in most cases. This is probably due to the higher number of parameters that need to be estimated when *M* increases. As for the *sit-stand-walk* dataset, the results in Table 4 seem to indicate a preference for values of *M* greater than one. However, this is only when the mechanism for rejecting spurious data is disabled; otherwise, *M* = 1 is the winning choice. In the following we only present results in the case that *M* = 1. 

### 3.1. The Seven-Activity Dataset

In Table 3, the classification accuracy, averaged across all tested classifiers, is reported. The estimated TPM turned out to be practically identical to the TPM specified in Table 1 to define virtual experiments. As far as the algorithm for rejection of spurious data is concerned, the threshold is fixed so as to achieve sensitivity (Se = 86.1%) and specificity (Sp = 86.7%.) The classification accuracy in the presence of spurious data and after their automatic rejection is presented for either the GMM or the cHMM. The confusion matrix obtained by HMM (1st + 2nd level) classification after spurious data rejection is reported in Table 5 results from all subjects are aggregated.

For some unknown reasons, we observe that three subjects are characterized by unusually low values of classification accuracy.  Table 6 shows the results when these subjects are not considered.

### 3.2. The Sit-Stand-Walk Dataset

**Figure 5:**
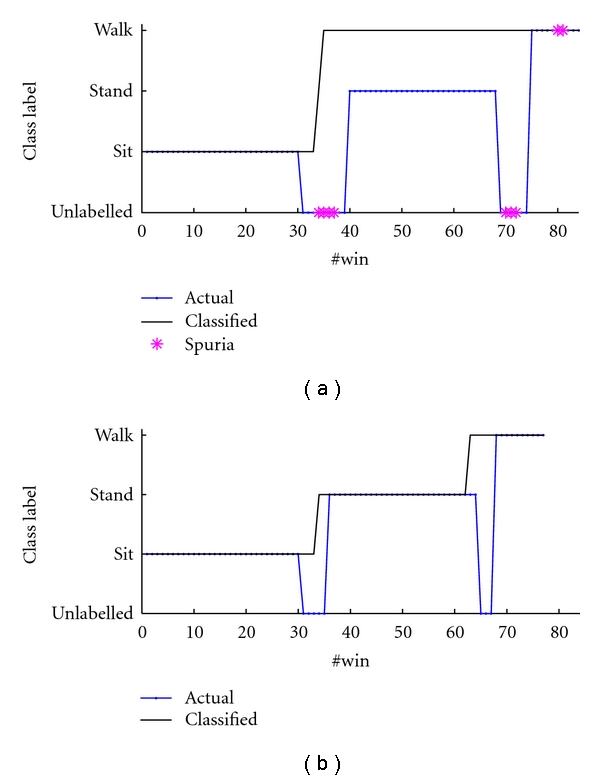
The method of spurious data rejection is shown in action (see text).

**Figure 6:**
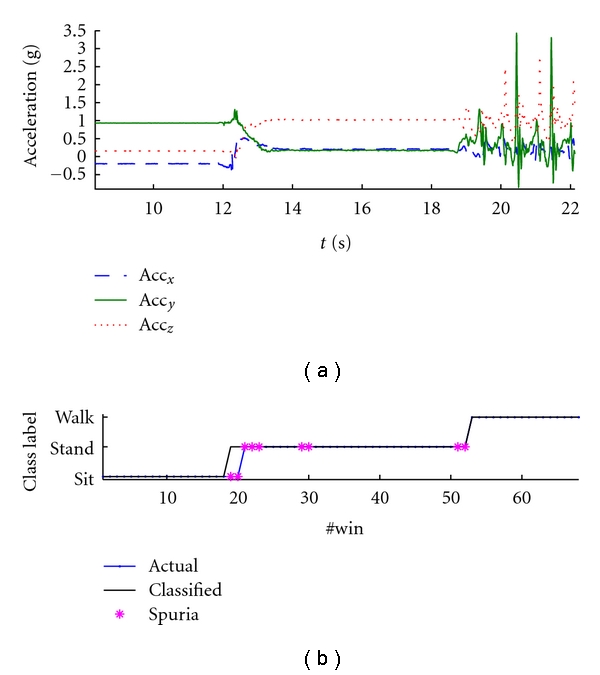
Classification and spurious data rejection on a sequence of the *sit-stand-walk* dataset.

The classification accuracy results are reported in Table 4 whereas the confusion matrix for the HMM classifier after rejection of spurious data is reported in Table 7. The spurious rejection algorithm is now applied to tag data with low reliability for classification. A higher number of tagged data is close to where activity transitions take place, Figure 6. Finally, Figure 5 describes the action of the spurious data rejection mechanism more in detail. The upper plot shows a typical example of a bad behavior of the Viterbi decoder close to a *sit*-to-*stand* transition when the rejection mechanism is turned off. A *sit*-to-*stand* transition is immediately followed by a *stand*-to-*walk* transition, which is wrong. Now, given that the cHMM emerges from training with a left-right structure, flying back to *stand* once the system enters *walk* is forbidden. As the lower plot shows, when the system decides not to decide in the presence of data frames of uncertain origin, the action of the Viterbi decoder may be more correct. This time, indeed, the *sit*-to-*stand* transition is correctly observed, and the system is ready to recognize the *stand*-to-*walk* quite close to when it actually occurs.

## 4. Discussions and Conclusions

Our decision to concentrate on a basic vocabulary of activities is motivated by our ongoing work aimed at developing a wearable sensor system for pedestrian navigation and human locomotion rehabilitation. Referring to the *seven-activity* dataset, the Pudil's feature selection scheme individuates a subset of features that simply consist of movement coordination information (correlation coefficients). Nonetheless, it is argued that DC component, energy, and entropy time-domain features would be highly valuable, provided that we decide to investigate other activities, for example, those from the set studied in [10] that are not considered in this paper. Although being limited to three activities chained in a fixed order, and lasting few seconds only, the tests on the *sit-stand-walk* dataset show that the proposed algorithm can also be applied to data in which activity transitions are naturalistic.

The generalization abilities of the tested classifiers can be considered good, since, for both datasets, a leave-one-out-subject validation strategy is adopted. Nonetheless, the classification accuracies are high, although the variability in terms of standard deviation is particularly high in the virtual experiments on the *seven-activity* dataset. This may be due to different factors: as compared with the *sit-stand-walk* dataset, a higher number of activities and subjects are considered in the *seven-activity* dataset, and, finally, the data contamination policy in the virtual experiments is quite aggressive. The main fact is, we believe, that some subjects may perform the same activities differently with respect to other subjects: as our data in Tables 2 and 4 clearly show, this is at odds with the generalization ability of tested classifiers.

The supervised training is pursued in this paper with the idea to split the process of estimating the parameters of the cHMM-based classifier into two distinct levels. The second-level training process is based on parameter reestimation using the Baum-Welch algorithm. In the results reported in this paper, the effects of the Baum-Welch algorithm are uncertain and of limited utility. However, the Baum-Welch algorithm is the classic approach for HMM parameter reestimation, even when a good initialization for supervised training is available [23, 28–31]. The rationale for parameter reestimation in a supervised framework is the possible improvement of generalization capabilities by the classifier at the expense of some reduction of its specificity. Some recent studies discuss an interesting approach where the Baum-Welch algorithm is modified in the attempt to make an HMM-based classifier adaptive [32]. We are currently investigating a modification of our proposed classifiers in this direction.

An interesting point in our approach is related to the proposed method for managing spurious feature vectors. Most published studies, including [10], handle the problem of the fuzzy borders by manual data cropping. Clearly this is neither useful nor applicable if we look for a real-time system for activity classification. In our approach, the whole spurious rejection process is made automatic. When one-third of the whole feature vectors in the data are spurious, such as in the virtual experiments described in the paper, the classification accuracies of the tested sequential classifiers are limited to about 64% in the absence of the proposed threshold-based detector. If the threshold-based detector is actually implemented, the performances ramp up to about 86%. When the spurious data cannot be tagged with certainty in advance, such as in the *sit-stand-walk* experiments, the performance boost provided by the threshold-based detector is not as impressive as in the virtual experiments however, we see that the cHMM benefits from a quite remarkable increment from 95% to 99%.

The cHMM-based classifier outperforms the GMM classifier by exploiting the statistical information of the activity dynamics. However, the statistical leverage of the HMM comes with its own problems. This is evident in analyzing the results obtained by working with the *sit-stand-walk* dataset. First, we observe that, in the *sit-stand-walk* experiments, on average, the performances of either the GMM or the cHMM are quite similar. However, in the absence of the threshold-based detector of spurious data, the cHMM tends to exhibit a more erratic behavior as compared with the GMM, see results in Table 5. Why is this so? The cHMM relies on data that it has to be considered emitted from its internal states, in other words, the Markov model must account for all possible observations in order to draw meaningful inferences. When something goes wrong, the cHMM tends to be stubborn in relying on its statistical memory, which is however wrong, as vividly shown in Figure 5. When a GMM classifier is considered, the emission models are the same as in the cHMM, but the GMM classifier does not pay regard to connections between states which are not modeled. Fortunately, when spurious data are prevented from affecting its behavior, the cHMM tends to perform better than the GMM classifier.

In conclusion, the applicability of Markovian modeling to the classification of human physical activities has been demonstrated. In particular, we have highlighted the importance of exploiting the statistical knowledge about the human motion dynamics that can be “trapped” within the Markov chain. The algorithm includes an effective device for rejecting spurious feature vectors, which turns out to show high sensitivity and specificity of detection.

## Figures and Tables

**Table 1:** TPM for the virtual experiments simulated in this paper (*seven-activity* dataset).

	*S*_1_	*S*_2_	*S*_3_	*S*_4_	*S*_5_	*S*_6_	*S*_7_
*S*_1_	0.95	0.20	0.00	0.00	0.00	0.01	0.04
*S*_2_	0.00	0.90	0.00	0.04	0.00	0.01	0.05
*S*_3_	0.00	0.00	0.62	0.25	0.01	0.02	0.10
*S*_4_	0.00	0.01	0.03	0.80	0.02	0.07	0.07
*S*_5_	0.00	0.01	0.01	0.35	0.40	0.01	0.22
*S*_6_	0.02	0.00	0.00	0.04	0.00	0.85	0.09
*S*_7_	0.01	0.03	0.01	0.18	0.03	0.12	0.62

**Table 2:** Classification accuracy averaged over the twenty subjects available in the *seven-activity* dataset. Spurious data are not inserted. The values are reported as mean ± standard deviation.

Seven-activity dataset (in the absence of spurious data)					
	*M* = 1	*M* = 2	*M* = 3	*M* = 4	*M* = 5
GMM	88.6 ± 13.7	88.1 ± 13.4	85.9 ± 13.6	82.5 ± 16.4	82.0 ± 16.6
cHMM (1st level)	90.2 ± 13.0	88.0 ± 12.9	85.6 ± 13.0	80.8 ± 17.1	79.3 ± 17.7
cHMM (1st + 2nd level)	90.2 ± 13.0	88.8 ± 13.4	85.2 ± 14.5	83.5 ± 16.3	82.8 ± 18.1

**Table 3:** Classification accuracy averaged over the twenty subjects available in the *seven-activity* dataset in the presence of spurious data. The values are reported as mean ± standard deviation.

Seven-activity dataset (spurious data present in proportion 1 : 3)					
	*M* = 1	*M* = 2	*M* = 3	*M* = 4	*M* = 5
Without rejection of spurious data					
GMM	63.3 ± 9.9	62.4 ± 11.4	60.2 ± 11.2	60.5 ± 13.0	56.0 ± 11.2
cHMM (1st level)	63.8 ± 9.3	61.4 ± 10.6	57.8 ± 10.9	55.0 ± 14.0	42.6 ± 13.9
cHMM (1st + 2nd level)	63.8 ± 9.3	61.8 ± 9.7	57.0 ± 12.5	50.1 ± 15.5	43.4 ± 14.6
With rejection of spurious data					
GMM	85.4 ± 13.4	86.7 ± 14.9	83.4 ± 15.3	80.5 ± 16.2	81.7 ± 17.6
cHMM (1st level)	86.2 ± 12.9	87.1 ± 13.7	83.0 ± 14.6	80.2 ± 15.7	81.9 ± 17.6
cHMM (1st + 2nd level)	86.2 ± 12.8	86.1 ± 13.3	83.2 ± 14.0	76.1 ± 16.7	75.9 ± 20.6

**Table 4:** Classification accuracy averaged over the seven subjects available in the *sit-stand-walk* dataset. The values are reported as mean ± standard deviation.

Sit-stand-walk dataset					
	*M* = 1	*M* = 2	*M* = 3	*M* = 4	*M* = 5
Without rejection of spurious data					
GMM	94.1 ± 1.8	93.7 ± 4.1	94.8 ± 2.4	95.5 ± 1.9	95.8 ± 2.1
HMM (1st level)	95.2 ± 7.0	96.8 ± 3.0	98.5 ± 1.0	98.3 ± 1.1	98.1 ± 1.1
HMM (1st + 2nd level)	95.2 ± 7.0	97.4 ± 2.9	97.9 ± 2.4	97.0 ± 2.9	98.5 ± 1.0
With rejection of spurious data					
GMM	94.0 ± 1.8	95.6 ± 1.5	95.8 ± 2.3	95.6 ± 1.7	95.6 ± 1.5
HMM (1st level)	99.0 ± 1.0	98.6 ± 1.0	98.8 ± 1.0	98.8 ± 1.1	98.6 ± 1.1
HMM (1st + 2nd level)	99.0 ± 1.0	98.8 ± 1.0	98.8 ± 1.0	98.7 ± 1.1	98.7 ± 1.0

**Table 5:** Confusion matrix obtained comparing HMM (1st + 2nd level) classifier output (columns) and the actual activity class labels after spurious data rejection (rows). All subjects' results are aggregated.

cHMM	*S*_1_	*S*_2_	*S*_3_	*S*_4_	*S*_5_	*S*_6_	*S*_7_
*S*_1_	9686	10	3	29	1	53	22
*S*_2_	0	6064	15	15	0	0	682
*S*_3_	0	29	1825	2543	25	0	921
*S*_4_	0	420	347	19189	35	0	613
*S*_5_	0	2	4	1	580	6	23
*S*_6_	2	0	0	0	0	18823	0
*S*_7_	0	293	47	376	3	0	9939

**Table 6:** Classification accuracy averaged over the seventeen subjects available in the *seven-activity* dataset, after removal of three anomalous subjects (see text). Spurious data are not inserted and *M* = 1.

Classifier	Accuracy
GMM	93.5 ± 6.0
cHMM (1st level)	95.1 ± 4.8
cHMM (1st + 2nd level)	95.1 ± 4.8

**Table 7:** Confusion matrix obtained comparing HMM (1st + 2nd level) classifier output (columns) and the actual activity class labels after spurious data rejection (rows). All subjects' results are aggregated.

cHMM	Sit	Stand	Walk
Sit	4973	10	0
Stand	0	3989	28
Walk	0	64	2270
